# Increase in Right Temporal Cortex Thickness Is Related to Decline of Overall Cognitive Function in Patients With Hypertension

**DOI:** 10.3389/fcvm.2021.758787

**Published:** 2021-11-25

**Authors:** Wei Li, Ling Yue, Shifu Xiao

**Affiliations:** ^1^Department of Geriatric Psychiatry, Shanghai Mental Health Center, Shanghai Jiao Tong University School of Medicine, Shanghai, China; ^2^Alzheimer's Disease and Related Disorders Center, Shanghai Jiao Tong University, Shanghai, China

**Keywords:** hypertension, cognition, elderly, longitudinal study, temporal pole, cortical thickness

## Abstract

**Background:** Hypertension is associated with poorer cognitive functions, but the mechanisms are unclear.

**Objective:** This research aims to explore the cognitive status of elderly patients with hypertension and the possible mechanisms of hypertension affecting cognitive function.

**Methods:** Data were obtained from the China Longitudinal Aging Study (CLAS), and a total of 128 residents, aged 60 years and above, were recruited in this study. Based on whether they had hypertension, these 128 people were divided into the hypertension (*n* = 64) and non-hypertension groups (*n* = 64). The Beijing version of the Mini-Mental State Examination (MMSE) and Montreal Cognitive Assessment (MoCA) were used to assess the overall cognitive function of the subjects, while digit span, language fluency, Wechsler mapping, and Wechsler wood block were used to assess their domain-specific cognitive function (both at baseline and follow-up stages). At the same time, we also examined baseline blood biochemical indicators (such as total protein, fasting plasma glucose (FPG), high-density lipoprotein (HDL), low-density lipoprotein (LDL), cholesterol, and triglyceride) and baseline MRI data of hippocampus and amygdala volume and temporal polar cortex thickness.

**Results:** The total protein and thickness of temporal polar cortex in patients with hypertension were significantly higher than those in normal controls, but the scores on MMSE, MoCA, digit span, Wechsler mapping and Wechsler wood block at baseline were significantly lower than those in normal controls (*p* < 0.05). By linear regression analysis and correlation analysis (age and education were controlled), we found that baseline Wechsler mapping scores were negatively correlated with total protein (*B* = −0.243, *t* = −3,735, *p* < 0.001, 95% confidence interval (CI): −0.371 to −0.114); and both the follow-up MMSE score (*B* = 2.657, *t* = 2.002, *p* = 0.049, 95% CI: 0.009~5.306) and the change score of MMSE (*r* = −0.025, *p* = 0.047) were related to the thickness of the right temporal pole cortex. Then, by linear regression analysis (mediating model), we found that hypertension may influence follow-up MMSE scores by influencing the cortical thickness of the right temporal pole (*B* = 1.727, *p* = 0.022, 95% CI: 0.261–3.193).

**Conclusions:** Elderly patients with hypertension exhibit poorer overall cognitive function and executive function, and the mechanism may be related to the effect of hypertension on the cortical thickness of the right temporal pole.

## Introduction

Hypertension is a very common disease that affects one third of adults in the world and two-thirds of adults over the age of 65 (1). It has been proved to be a risk factor for the development of cognitive decline, vascular dementia (VD), and Alzheimer's disease (AD) (2). Intermediate-quality evidence suggests that hypertension in middle age is associated with a 1.19- to 1.55-fold risk of cognitive impairment (3), and the overall pooled prevalence of mild cognitive impairment (MCI) in patients with hypertension is 30% (95% CI, 25–35) (4), while the prevalence of MCI in the general population is only 16% (5). The association between dementia and mortality can be influenced by hypertension (coefficient −0.009, *p* = 0.02) (6), and there is the vast epidemiologic and mechanistic evidence linking the positive effects of blood pressure lowering on reducing the risk of post-stroke dementia and other types of dementia (7, 8).

However, there is little neuro-basic evidence for the relationship between hypertension and cognitive deterioration. Since the correlation between morphological and neuropsychological characteristics of the brain is well-known, hypertension may be related to changes in specific brain structures throughout life (9). It has been reported that the key brain structures for hypertension include cerebral white matter (10), hippocampal volume (11), cerebral gray matter (12), medial, temporal (13), posterior cingulate, and orbitofrontal (14). The temporal pole is a part of the para-limbic system along with the insula, orbitofrontal cortex, anterior cingulate cortex, and other emotion-related regions (15). Damage to the temporal pole, which plays a key role in social and emotional processing, can lead to unstable emotional states (16). In the study by M O'Sullivan, they found that temporal pole hyper-intensity is a radiologic marker of cerebral autosomal dominant arteriopathy with subcortical infarcts and leukoencephalopathy (17). Shikimoto et al. (18) found that the resilience capacity might be related to temporal cortices in older adults. However, few studies have explored the relationship between hypertension and the temporal pole.

To fill in the gaps in current research, we focused on the relationship between hypertension, temporal polar cortex thickness [previous studies have shown that cortical thickness is more susceptible to age and memory function than brain structure (19)] and cognitive function. Our hypothesis is that the cognitive function of patients with hypertension is worse than that of normal controls, and that hypertension may affect cognitive function through temporal polar cortical thickness.

## Materials and Methods

### Participants

One hundred twenty-eight elderly (male/female = 62/66) with normal cognition were recruited from the community. All the participants met the following requirements: (1) aged 60 or older; (2) with normal cognitive ability; (3) without severe medical condition such as cancer and infections; (4) without serious mental illness such as schizophrenia and severe depression; (5) without cognitive-related diseases such as dementia and mild cognitive impairment; (6) be willing to cooperate. Using standardized questionnaires, we collected the general demographic data of the subjects [such as gender, age, education, body mass index (BMI), systolic blood pressure, and diastolic blood pressure], daily living information (such as smoking, drinking alcohol, tea), and disease-related information (such as hypertension and diabetes).

The study protocol was endorsed by the Research Ethical Committee of the affiliated mental health center of Shanghai Jiao Tong University School of Medicine. Written informed consent was issued by all the participants before the study.

### Measurement of Hypertension

Hypertension status was based on self-reported diagnosis of a physician or treatment with an oral anti-hypertensive drug. Individuals are also considered to have hypertension if their mean systolic blood pressure (SBP) ≥ 140 mm Hg and/or diastolic blood pressure (DBP) ≥ 90 mmHg (20). Based on these criteria, 64 of the 128 were diagnosed with hypertension, while the remaining 64 were considered normal controls.

### MR Image Acquisition and Processing

A brain structure image was acquired using a Siemens Magnetom Verio 3.0 T scanner (Siemens, Munich, Germany). The whole acquisition time was 8 min and 7 s. If there are any pathological findings, the image is examined and discarded. The parameters of T1-weighted 3D magnetization prepared rapid gradient echo (MPRAGE) sequences were as follows: TE = 2.98 ms, TR = 2,300 ms; matrix size = 240 × 256; flip angle of 9 degrees, field of view (FOV) = 240 mm × 256 mm; slice thickness = 1.2 mm. Volumetric data were assessed by automated procedures, which have been described by Wolz et al. (21). Cortical thickness and cortical volume for each individual was extracted directly using FreeSurfer v6.0 (surfer.nmr.mgh.harvard.edu), and the processing steps were skull stripping, spatial transformation, atlas registration, surface reconstruction, spherical surface mapping, and atlas-based regional parcellation. Quality control was performed by superimposing output slices on the FreeSurfer template, and visual evaluation was performed to ensure registration and slice quality.

### Blood Biochemical Index Detection

All the subjects stopped eating after 9 pm, and their peripheral blood was collected between 7:00 and 9:00 a.m. (the next morning). Anticoagulant tubes and clot activating gel-containing serum separator tubes were used to assay blood indexes, such as the total protein, fasting plasma glucose (FPG), high-density lipoprotein (HDL), low-density lipoprotein (LDL), cholesterol, and triglyceride.

### Neuropsychological Tests

All the participants had received a detailed physical examination, clinical evaluation, and neuropsychological tests. The Beijing version of the Mini-Mental State Examination (MMSE) and Montreal Cognitive Assessment (MoCA) were used to assess the overall cognitive function of the subjects, while digit span, language fluency, Wechsler mapping, and Wechsler wood block were used to assess their domain-specific cognitive function.

#### Mini-Mental State Examination (MMSE)

Currently, the MMSE is the most commonly used cognitive function assessment scale (22). The MMSE can assess a wide range of domains, such as memory, orientation, attention, language, and visuospatial proficiency, and its sensitivity and specificity for detecting dementia are 88.3 and 86.2%, respectively (23). However, it is less sensitive to MCI detection, susceptible to, education level and, due to copyright restrictions, use of the scale may require payment (24).

#### Montreal Cognitive Assessment (MoCA)

The Beijing version of MoCA is a brief screening test for cognitive impairment that covers major cognitive domains, such as memory, attention, orientation, language, visuospatial ability, and executive functions (22). MoCA has been proved to be effective in distinguishing AD, MCI, and normal elderly (25), and can detect 90% of subjects with MCI, with high sensitivity and specificity (26). Compared with MMSE, the MoCA scale shows a higher sensitivity (27). Under the recommended cut-off score of 26, the Beijing version of MoCA demonstrated an excellent sensitivity of 90.4, and fair specificity (31.3) (28).

#### Digit Span

The Digit span test includes both digit forward and digit backward conditions, and is widely performed to assess working memory and attention-concentration (29). It is portable, quick, and easy to use, often forming an integral part of the mental state examination and neuropsychological examinations. Good performance on the Digit span test requires short-term verbal memory and auditory attention, and the integrity of the left hemisphere is more important than the right hemisphere or diffuse damage (30).

#### Language Fluency

Verbal fluency refers to the ability to speak fluently. The measurement of phoneme and semantic fluency, such as the controlled oral word association test (COWAT, also known as oral fluency or VF), is usually conceptualized as measuring executive function (31). Previous studies have suggested that general verbal fluency is also strongly associated with word knowledge, auditory attention, and lexical ability and access (32). In the current study, two forms of fluency were used: phonemic (that is, letter oriented) and semantic (that is, category oriented), where, for a limited time, the candidates were asked to provide answers that began with a particular letter or that fit a target category. A higher score for verbal fluency was associated with better executive performance.

#### Wechsler Mapping and Wechsler Woodblock

Neuropsychological tests of executive function were performed using by Wechsler mapping and Wechsler wood block. Lower scores for Wechsler mapping and Wechsler wood block indicate more severe impairment of executive function (33).

### Study Design

All the subjects completed clinical evaluation, neuropsychological test evaluation, blood biochemical test, and structural magnetic resonance test at the baseline stage. However, we only carried out clinical evaluation and neuropsychological test evaluation, and did not carry out blood biochemical and structural magnetic resonance tests at the follow-up of 1 year.

### Statistical Analysis

Continuous variables were expressed as mean ± SD, and categorical variables were expressed as frequencies (%). A single-sample Kolmogorov–Smirnov test was performed to test whether the data conformed to normal distribution. Mann–Whitney tests or independent sample *t*-tests were performed to compare non-normally distributed data and normally distributed data between the hypertension group and the normal control group, respectively. A Chi-square test was performed to compare categorical variables between the two groups. A linear regression analysis (mediating model) and a correlation analysis were performed to investigate the association among hypertension, cognitive-related brain areas, blood biochemical index, and cognitive scores (age and education were controlled). All the statistical analyses were performed using SPSS 22.0 (IBM Corporation, Armonk, NY, United States), and two-tailed tests were performed at a significance level of *P* < 0.05.

## Results

### Characteristics of Study Patients

Total protein, SBP, and thickness of temporal polar cortex in patients with hypertension were significantly higher than those in normal controls, but the scores on MMSE, MoCA, digit span, Wechsler mapping, and Wechsler wood block at baseline were significantly lower than those in normal controls (*p* < 0.05), and there was no statistical difference (*p* > 0.05) in age, education, BMI, diastolic blood pressure, male, smoker, alcohol drinker, tea drinker, physical exercise, hobby, diabetes, fasting plasma glucose, triglycerides, cholesterol, HDL, LDL, total brain volume, left amygdala, right amygdala, left hippocampus, right hippocampus, language fluency score at baseline, and scores on MMSE, MoCA, digit span, language fluency, Wechsler mapping, and Wechsler wood block at 1-year follow-up between the two groups. Table 1 presents the results.

**Table 1:** General demographic data between the two groups.

**Variables**	**Hypertension (*n* = 64)**	**Non-hypertension (*n* = 64)**	***X*^2^ or t**	***p***
Age, y	70.09 ± 7.177	70.59 ± 8.110	−0.369	0.712
Education, y	9.41 ± 3.942	9.31 ± 4.222	0.145	0.885
BMI, kg/m^2^	24.64 ± 3.368	24.16 ± 3.892	0.579	0.564
Systolic blood pressure, mmHg	137.66 ± 15.355	126.28 ± 14.799	4.267	<0.001^*^
Diastolic blood pressure, mmHg	80.03 ± 9.780	77.31 ± 10.106	1.547	0.124
Male, *n* (%)	29 (45.3)	33 (51.6)	0.500	0.596
Smoker, *n* (%)	18 (28.1)	17 (26.6)	0.039	1.000
Alcohol drinker, *n* (%)	9 (14.1)	13 (20.3)	0.878	0.483
Tea drinker, *n* (%)	23 (35.9)	23 (35.9)	0.000	1.000
Physical exercise, *n* (%)	43 (67.2)	42 (65.6)	0.035	1.000
Hobby, *n* (%)	40 (62.5)	46 (71.9)	1.276	0.347
Diabetes, *n* (%)	0	0	-	–
**Blood biochemical index**
The total protein, g/L	73.54 ± 4.095	71.68 ± 5.609	2.062	0.041^*^
Fasting plasma glucose, mmol/L	4.87 ± 0.909	4.86 ± 0.698	0.088	0.930
Triglycerides, mmol/L	1.63 ± 0.871	1.57 ± 1.536	0.271	0.787
Cholesterol, mmol/L	4.84 ± 1.058	4.96 ± 1.174	−0.581	0.562
High-density lipoprotein, mmol/L	1.18 ± 0.332	1.26 ± 0.360	−1.275	0.205
Low density lipoprotein, mmol/L	3.10 ± 0.780	3.19 ± 1.066	−0.515	0.608
**Ti-baseline structural MRI**
Total brain volume, mm^3^	1454894.67	1456356.13	−0.047	0.962
Left Amygdala, mm^3^	1486.74 ± 232.52	1504.72 ± 236.83	−0.433	0.665
Right Amygdala, mm^3^	1690.28 ± 264.66	1608.19 ± 273.75	1.725	0.087
Left hippocampus, mm^3^	3589.89 ± 437.86	3588.38 ± 455.15	0.019	0.985
Right hippocampus, mm^3^	3744.87 ± 456.11	3761.05 ± 581.85	−0.175	0.861
Left temporal pole thickness, mm^3^	3.48 ± 0.247	3.38 ± 0.294	2.035	0.044^*^
Right temporal pole thickness, mm^3^	3.54 ± 0.269	3.42 ± 0.302	2.382	0.019^*^
**Baseline neuropsychological tests**
MMSE	27.32 ± 2.271	28.14 ± 1.959	−2.189	0.030^*^
MoCA	23.27 ± 4.147	25.05 ± 4.025	−2.450	0.016^*^
Digit span	13.89 ± 4.221	15.78 ± 4.077	−2.578	0.011^*^
Language fluency	26.75 ± 7.947	29.71 ± 10.629	−1.775	0.078
Wechsler mapping	9.83 ± 3.861	11.50 ± 4.324	−2.307	0.023^*^
Wechsler wood block	25.87 ± 7.343	29.14 ± 9.263	−2.196	0.030^*^
**Follow-up neuropsychological tests**
MMSE (follow)	26.38 ± 3.756	27.75 ± 2.356	−1.827	0.072
MoCA (follow)	22.68 ± 5.474	23.69 ± 4.687	−0.849	0.398
Digit span (follow)	16.02 ± 15.500	15.50 ± 3.810	0.186	0.853
Language fluency (follow)	27.21 ± 8.059	25.28 ± 10.337	0.932	0.354
Wechsler mapping (follow)	9.28 ± 4.287	10.88 ± 3.661	−1.723	0.089
Wechsler woodblock (follow)	26.17 ± 6.917	26.48 ± 10.347	−0.161	0.873

* *Means p <0.05*.

### Influencing Factors of Baseline Cognitive Scores

By the single-sample Kolmogorov-Smirnov test, we found that the baseline scores on MOCA, digit span, Wechsler mapping, and Wechsler woodblock accorded with normal distribution, while the baseline scores on MMSE did not accord with normal distribution. Therefore, the linear regression analysis (baseline MOCA score, digit span score, Wechsler mapping and Wechsler wood block) and correlation analysis (baseline MMSE score) were performed to explore the relationship between baseline cognitive scores and total protein and temporal polar cortex thickness (age and education were controlled). Finally, we found that baseline Wechsler mapping scores were negatively correlated with total protein (*B* = −0.243, *t* = −3.735, *p* < 0.001, 95% CI: −0.371 to −0.114), while the other baseline cognitive scores were not correlated with (*p* > 0.05) total protein or temporal polar cortical thickness. Table 2 presents the results.

**Table 2:** Influencing factors of baseline Wechsler mapping (linear regression analysis).

**Variables**	***B***	***t***	***p***	**95% confidence interval**
The total protein	−0.234	−3.735	<0.001^*^	−0.371~-0.114
Left temporal pole thickness	−0.082	−0.069	0.945	−2.424~2.260
Right temporal pole thickness	−1.482	−1.305	0.194	−3.729~0.766

* *Means p <0.05*.

### Influencing Factors of Follow-Up Cognitive Scores

**Figure 1:**
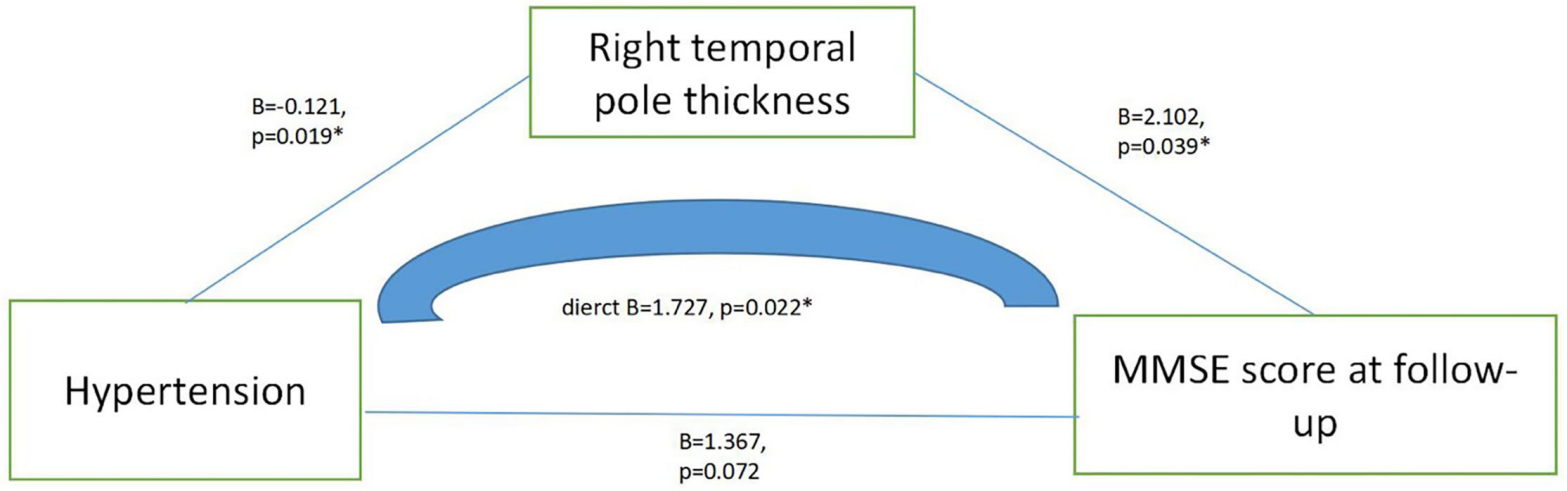
Mediating effect model among hypertension, right temporal pole thickness and MMSE score at follow-up.

Through the linear regression analysis (with follow-up MMSE score as dependent variable, total protein, baseline MMSE score, left temporal pole cortex thickness and right temporal pole cortex thickness as independent variables, age and education were controlled), we found that follow-up MMSE score was related to right temporal pole cortex thickness (*B* = 2.657, *t* = 2.002, *p* = 0.049, 95% CI: 0.009–5.306). Table 3 presents the results. By the linear regression analysis (mediation model), we found that hypertension directly affected the MMSE score during follow-up by affecting the thickness of the right temporal pole cortex (*B* = 1.727, *p* = 0.022, 95% CI: 0.261–3.193). Figure 1 presents the results.

**Table 3:** Influencing factors of follow-up Mini-Mental State Examination (MMSE) scores (linear regression analysis).

**Variables**	***B***	***t***	***p***	**95% confidence interval**
The total protein	0.084	1.467	0.147	−0.030~0.199
Left temporal pole thickness	−1.140	−0.892	0.376	−3.693~1.412
Right temporal pole thickness	2.657	2.002	0.049^*^	0.009~5.306
Baseline MMSE score	0.898	7.108	<0.001^*^	0.646~1.150

* *Means p <0.05*.

### Influencing Factors of Cognitive Change Scores (Baseline Cognitive Score—Follow-Up Cognitive Score)

**Figure 2:**
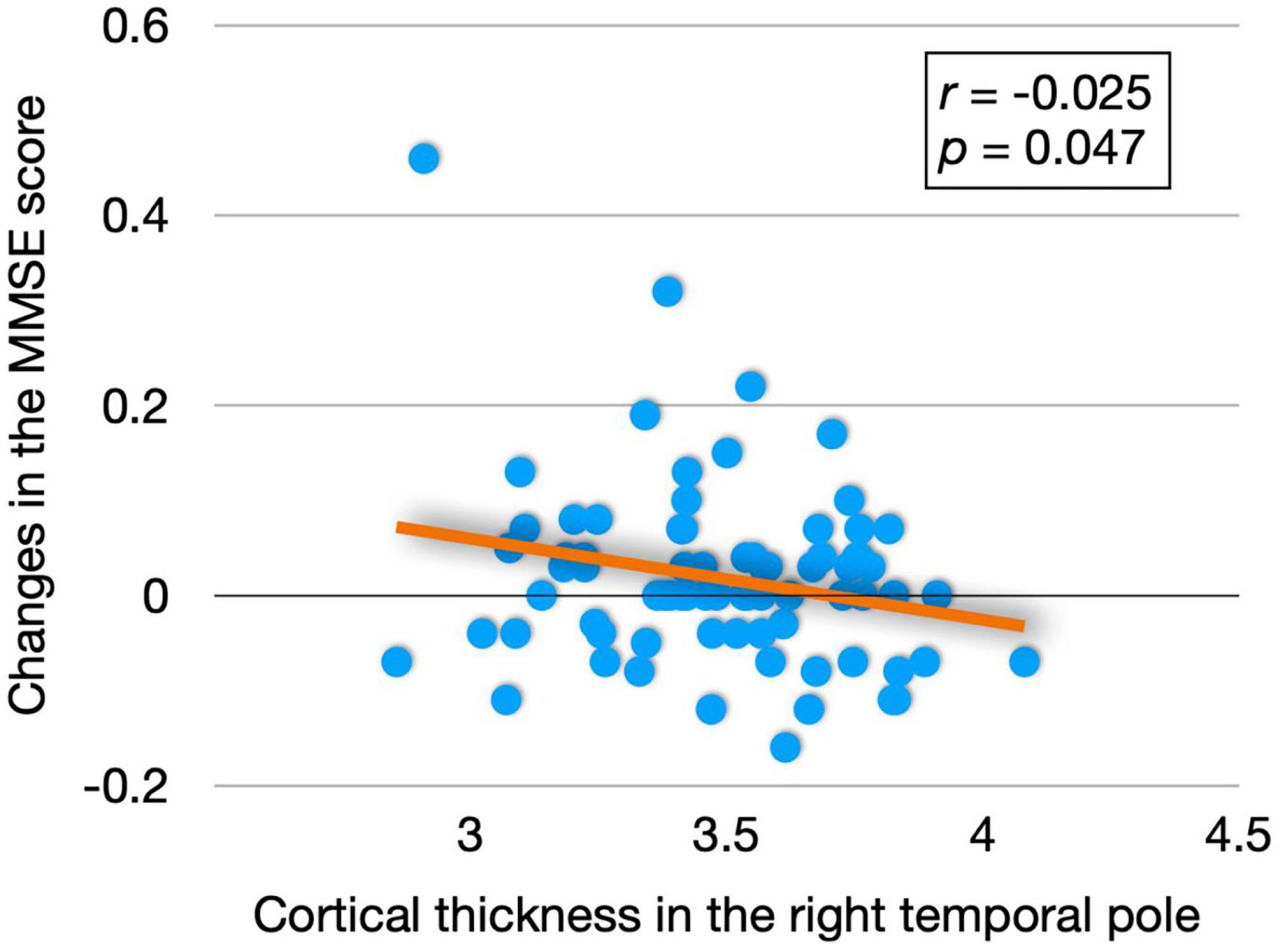
Relationship between MMSE score and right temporal pole cortical thickness.

The partial correlation analysis (age and education were controlled) showed that there was a significant negative correlation between the cortical thickness of the right temporal pole and change in the MMSE score (*r* = −0.025, *p* = 0.047), Figure 2 presents the results.

## Discussions

The purpose of this study was to explore the state of cognitive function in elderly patients with hypertension and the possible mechanism of hypertension regulating cognitive function, and we found that: (1) the elderly with hypertension showed worse overall cognitive function and executive dysfunction; (2) there was a significant negative correlation between total protein and executive function; (3) the cortical thickness of the right temporal pole played a fully mediating role in the effect of hypertension on cognitive function.

Hypertension is one of the most important modifiable risk factors for cardiovascular and cerebrovascular diseases, which can lead to serious target organ damage. Studies have shown that hypertension is related to the increased prevalence of cognitive decline (34, 35). For example, Li et al. (36) found that higher long-term blood pressure variability was associated with accelerated cognitive decline in general adults aged ≥ 50 years in a non-linear dose-response relationship. Ungvari et al. pointed out that hypertension could significantly increase the risk of both Alzheimer's disease and vascular cognitive impairment (37). In our study, we used a range of scales to assess the cognitive function of the subjects, both overall and dome-specific. Not surprisingly, we found that the hypertensive patients exhibited poorer overall cognitive status and poorer executive function status. Wei et al. (38) found that systolic blood pressure (SBP) and pulse pressure (PP) were significantly negatively correlated with cognitive function in people over 60 years old. Naharci et al. (39) found that blood pressure index (BPI) was associated with cognitive ability and might be a new alternative marker for identifying dementia risk in older adults. What is more, in the study of Natália Cristina Moraes, they also found that patients with systemic arterial hypertension (SAH) had a significant impairment in executive functions (EFs), more specifically in shifting and updating (40). Therefore, our conclusions were consistent.

Serum total protein, which can be divided into albumin and globulin, has important physiological functions (such as maintaining normal colloid osmotic pressure and pH of blood, transporting various metabolites, and regulating the physiological effect of transported substances) in the body. Serum total protein (TP) can be used to monitor the nutritional status of the body, and for the diagnosis and differential diagnosis of different diseases. Therefore, determination of TP is one of the important items in clinical biochemistry (41). Previous studies have shown that serum TP is strongly associated with hypertension. For example, Song et al. (42) found that serum total protein (TP) was an important predictive marker of depression in hypertension. Wu et al. (43) found that serum TP was statistically significantly related to systolic blood pressure (SB). Mone et al. (44) pointed out that hyperglycemia and hyperlipidemia may lead to cognitive decline by affecting endothelial cell function. What is more, Morys et al. found that obesity was associated with poor cognitive ability, and that obesity and metabolic consequences of cerebrovascular diseases were potential mediating factors (45). In our study, we also found that the serum TP content of hypertensive patients was significantly higher than that of normal controls, and that it was negatively correlated with executive function. Therefore, our findings were partially consistent.

To further explore the possible mechanisms by which hypertension affects cognitive functions, we then included magnetic resonance data. Based on the previous research, we mainly focused on the effects of hippocampal, amygdala, and temporal polar cortex thickness on cognitive function (46). We found that the cortical thickness of the left temporal pole and right temporal pole in hypertensive patients was significantly higher than that in normal controls. By the linear regression analysis (mediation model), we further found a negative correlation between the cortical thickness of the right temporal pole and MMSE score at follow-up, and that hypertension might affect cognitive function by affecting right temporal pole cortical thickness. Beason-Held et al. (47) found that high blood pressure could alter the activity patterns of the temporal polar cortex. However, Gonzalez et al. (48) found that hypertension was associated with thinning of cortical thickness in cognitive brain areas. Since we did not re-examine the head MRI of the subjects during the follow-up period, we were unable to determine the longitudinal effect of hypertension on temporal polar cortical thickness.

We have to admit that our study has some limitations. First, the sample size is relatively small, which will reduce the reliability of the study. Second, we did not obtain cranial magnetic resonance data of the subjects after follow-up, so it is impossible to further determine the effect of hypertension on temporal polar cortex thickness. Third, our follow-up period was too short (only 1 year), which might miss more meaningful brain areas.

## Conclusions

Elderly patients with hypertension exhibit poorer overall cognitive function and executive function, and the mechanism may be related to the effect of hypertension on the cortical thickness of the right temporal pole.

## Data Availability Statement

The original contributions presented in the study are included in the article/supplementary material, further inquiries can be directed to the corresponding author/s.

## Ethics Statement

The studies involving human participants were reviewed and approved by the Research Ethical Committee of the affiliated mental health center of Shanghai Jiaotong University School of Medicine. The patients/participants provided their written informed consent to participate in this study. Written informed consent was obtained from the individual(s) for the publication of any potentially identifiable images or data included in this article.

## Author Contributions

WL and LY contributed to the study concept and design. SX analyzed the data and drafted the manuscript. WL directed the analysis and statistics of MRI data. All the authors have read and approved the final version of the manuscript.

## Funding

This study was supported by grants from the clinical research center project of Shanghai Mental Health Center (CRC2017ZD02), Shanghai Clinical Research Center for Mental Health (19MC1911100), the Cultivation of Multidisciplinary Interdisciplinary Project in Shanghai Jiaotong University (YG2019QNA10), curriculum reform of Medical College of Shanghai Jiaotong University, and the Feixiang Program of Shanghai Mental Health Center (2020-FX-03). This project was also funded by the Shanghai Elderly Brain Health Cohort Institute, Chinese Academy of Sciences (XDA12040101), Shanghai Clinical Research Center for Mental Health (SCRC-MH, 19MC1911100), the National Natural Science Foundation of China (82001123), and the Shanghai Science and Technology Committee (20Y11906800).

## Conflict of Interest

The authors declare that the research was conducted in the absence of any commercial or financial relationships that could be construed as a potential conflict of interest.

## Publisher's Note

All claims expressed in this article are solely those of the authors and do not necessarily represent those of their affiliated organizations, or those of the publisher, the editors and the reviewers. Any product that may be evaluated in this article, or claim that may be made by its manufacturer, is not guaranteed or endorsed by the publisher.
